# Reproductive neuroendocrine pathways as modulators of sleep‐related sex differences in Alzheimer's disease

**DOI:** 10.1002/alz.71699

**Published:** 2026-07-28

**Authors:** Maingredy Rodrigues Souza, Tathiana A Alvarenga, Renata Mazaro‐Costa, Sergio Tufik, Monica L Andersen

**Affiliations:** ^1^ Instituto do Sono Associação Fundo de Incentivo à Pesquisa (AFIP) São Paulo Brazil; ^2^ Departamento de Psicobiologia Universidade Federal de São Paulo São Paulo Brazil; ^3^ Departamento de Farmacologia Universidade Federal de Goiás Goiânia Brazil

1

Dear Editor,

Sleep disturbances are increasingly recognized as contributors to Alzheimer's disease (AD) pathophysiology, yet their mechanistic role in disease progression remains incompletely defined. Wear et al.^1^ reported that chronic sleep disruption accelerated AD‐related neuropathology in a sex‐dependent manner in amyloid precursor protein (APP)^NL‐G‐F^ knock‐in mice. Particularly striking was the greater hippocampal neurodegeneration and inflammatory response observed in female mice, positioning sleep disruption as a mechanistically relevant and potentially modifiable contributor to AD progression.

Beyond strengthening the bidirectional link between sleep loss and impaired proteostasis,^2^ these findings highlight the role of sex‐specific neurobiological contexts in shaping vulnerability to sleep‐related AD mechanisms. Of note, they underscore the need to more fully incorporate sex as a biological variable in AD research. Women account for nearly two in three dementia cases worldwide.^3^ Sleep disturbances are highly prevalent in older adults and more frequent in women than in men.^4^ The historical underrepresentation of female participants in preclinical and clinical research has limited progress in identifying sex‐specific pathways involved in disease susceptibility and progression.^3^ We propose that reproductive neuroendocrine status may represent a previously underappreciated biological dimension through which sleep disruption differentially accelerates AD pathogenesis across the lifespan.

In this setting, sex‐dependent responses to sleep disruption may reflect integrated neuroendocrine pathways linking stress and reproductive function. In addition to activating the hypothalamic‐pituitary‐adrenal axis, sleep loss disrupts hypothalamic‐pituitary‐gonadal signaling, altering gonadal steroid balance in both sexes.^5^, ^6^ These endocrine shifts may partially explain why identical sleep insults produce distinct neurodegenerative and inflammatory phenotypes in male and female individuals.

Supporting this view, sex hormones modulate several pathways implicated in sleep‐related neurodegeneration. Estrogens exert broad neuroprotective effects on synaptic plasticity, neuronal survival, mitochondrial function, and inflammatory regulation, and may attenuate AD‐related pathology by reducing amyloid beta (Aβ) production and tau hyperphosphorylation. Estrogen signaling is closely linked to sleep regulation across brain regions involved in both sleep–wake control and AD vulnerability. Although less extensively studied, progesterone may likewise contribute to sleep regulation and neural stability in a context‐dependent manner.^3^

This perspective may be particularly relevant during the menopausal transition, a critical female vulnerability window in which ovarian hormone decline, sleep disruption,^7^ and AD biomarker burden may converge. In this phase, sleep may act as a mediator of the relationship between hormonal fluctuations and memory processes, with intra‐individual hormonal variability potentially shaping vulnerability to AD across the female lifespan.^8^ By contrast, the slower androgen decline in aging men may contribute to a distinct temporal trajectory of sleep‐related AD vulnerability.^9^, ^10^ Because the study was conducted in a 6‐month‐old mouse model, the observed sex‐dependent effects were examined in an adult, rather than endocrinologically aged, condition. Extending this line of investigation to aged animals or models of reproductive endocrine transition may help clarify whether these interactions become even more pronounced under conditions that more closely resemble human aging.

These observations suggest that reproductive neuroendocrine status may be an important modifier of susceptibility to sleep‐related AD mechanisms and deserves more systematic attention in translational research. Clinically, this supports sex‐informed strategies that integrate sleep assessment, circadian evaluation, and endocrine characterization, particularly during hormonally dynamic periods such as the menopausal transition. Rather than being viewed solely as a consequence of neurodegeneration, sleep disturbance may represent a modifiable target for earlier and more individualized intervention. Future longitudinal and interventional studies integrating sleep, circadian biology, and reproductive neuroendocrine profiling may help identify sex‐specific windows for precision prevention in AD.

## AUTHOR CONTRIBUTIONS

**Maingredy Rodrigues Souza**: Conceptualization; investigation; and writing — original draft. Tathiana A Alvarenga: supervision and review & editing. **Renata Mazaro‐Costa**: Review & editing. **Sergio Tufik**: Supervision; funding acquisition; and resources. **Monica L Andersen**: Conceptualization; supervision; review & editing; funding acquisition; and resources.

## CONFLICT OF INTEREST STATEMENT

The authors report no conflicts of interest. Author disclosures are available in the Supporting Information.

## FUNDING INFORMATION

This work was supported by the Associação Fundo de Incentivo à Pesquisa, São Paulo, Brazil. S.T. and M.L.A. received fellowships from the Conselho Nacional de Desenvolvimento Científico e Tecnológico. M.L.A. received a grant from the Fundação de Amparo à Pesquisa do Estado de São Paulo [grant number 2020/13467‐8].

## Supporting information



Supporting Information
